# Conceptualizing the Human Health Outcomes of Acting in Natural Environments: An Ecological Perspective

**DOI:** 10.3389/fpsyg.2020.01362

**Published:** 2020-07-16

**Authors:** Eric Brymer, Duarte Araújo, Keith Davids, Gert-Jan Pepping

**Affiliations:** ^1^Australian College of Applied Psychology, Brisbane, QLD, Australia; ^2^CIPER, Faculdade de Motricidade Humana, Universidade de Lisboa, Lisbon, Portugal; ^3^Human Performance and Sport, Sheffield Hallam University, Sheffield, United Kingdom; ^4^School of Behavioural and Health Sciences, Australian Catholic University, Brisbane, QLD, Australia

**Keywords:** ecological psychology, affordances, niche construction, form of life, natural environments, health and wellbeing

## Abstract

Human-nature interactions have been presented as important for promoting and sustaining wellbeing and health benefits. Research has shown that pictures of nature, interacting with nature, physical activity in nature, immersion in nature and even feeling connected to nature can improve health. While considerable research supports this notion that nature can have positive health impact, theoretical and conceptual frameworks that help explain *how* the natural environment provides benefits to human health and wellbeing have proved limited. In extreme cases, theoretical approaches reinforce a problematic notion where nature is viewed as a separate entity, as a treatment to be taken as prescribed to remediate health problems that arise. Such approaches are limited as they fail to address how beneficial person-nature relations may be captured in interventions. There is a need for a deeper understanding of the processes underlying the observed benefits of the person-nature link in order to design effective research and interventions. It is especially important to consider the implications of research on person-nature relations for people living in urban contexts. In this paper, we present an ecological perspective building on James Gibson’s conceptualization of human behavior. Specifically, we outline a framework that emphasizes the person-environment system as the most appropriate scale of analysis. We present three relevant concepts from the ecological approach: form of life, affordances and niche construction, as helpful for appreciating how acting in natural environments might benefit human health and wellbeing. This approach urges policy makers and urban designers to rethink environmental designs to provide and support a landscape of affordances that makes use of the richness of natural environments.

## Introduction

There is now a robust body of evidence suggesting that the relationship between human beings and nature can support positive health and wellbeing. Pictures of nature, interacting with nature, physical activity in nature, immersion in nature and feeling connected to, or part of, nature can improve health (Kaplan and Kaplan, 1989; Hartig et al., 1991; Ulrich et al., 1991; Kuo, 2001; Peacock et al., 2007; Tsunetsugu et al., 2009; Ryan et al., 2010; Roe and Aspinall, 2011; Berman et al., 2012; Wolsko and Hoyt, 2012; Gladwell et al., 2013; Berto, 2014; Passmore and Howell, 2014; Martyn and Brymer, 2016; Menatti and Casado da Rocha, 2016; Yeh et al., 2016; Lawton et al., 2017; Schweitzer et al., 2018). As a consequence, there is an increased interest in how to design nature-based activities and environments to enhance human and planetary health and wellbeing (Brymer et al., 2014; Brymer and Davids, 2016; Davids et al., 2016; Yeh et al., 2016,2017; Houge Mackenzie and Brymer, 2018). Despite the extensive practical work and empirical research demonstrating the positive relationship between experiences with nature and enhanced health and wellbeing, the conceptual and theoretical underpinnings of *how* person-natural environment relations can support health and wellbeing is limited (Bowler et al., 2010; Brymer et al., 2014; Brymer and Davids, 2016; Araújo et al., 2019a). Here, we argue that, for the most part, this deficit has emerged because traditional theoretical perspectives view nature and people as distinct and separate entities (Heft, 2012). Such a dualism has promoted a biased understanding of human behavior which is unlikely to help further an understanding of the processes supporting human-nature relations, or support effective health-focused research and intervention designs that are based around human-nature relations. In this paper, we draw on an ecological perspective building on Gibson’s (1979) work. Specifically, we propose a “transactional” framework that emphasizes the animal-environment relationship as the appropriate scale of analysis for understanding the health benefits (human and nature) from the human-nature system. We present three particular concepts from this ecological approach - form of life, affordances and niche construction - as helpful for appreciating how human-nature interactions might benefit health and wellbeing.

## Traditional Theories Explaining the Nature – Health and Wellbeing Relationship

Theoretical models explaining the relationship between nature experiences and health and wellbeing have predominately focused on explaining the mental health benefits gained from nature. The two main approaches emphasize either: 1. (physical) activity in nature; or 2. feeling connected to nature. For instance, Ulrich’s Psychoevolutionary Theory (PET; Ulrich, 1981), also known as stress reduction theory, proposes that humans prefer natural environments that provide safety and resource availability (e.g., water, shelter and vegetation). Attention Restoration Theory (ART; Kaplan and Kaplan, 1989) proposes that everyday human experiences in urban societies are cognitively taxing, requiring high levels of sustained effortful attention. Conversely, nature provides opportunities to restore attention. Finally, Nature Connection theories, such as, Nature Connectedness (NC) and Nature Relatedness (NR; Cervinka et al., 2012; Capaldi et al., 2014; Zelenski and Nisbet, 2014), are based on an individual difference narrative in which wellbeing is correlated with emotional connection with the natural environment (Mayer and Frantz, 2004), conceived as a mental state.

While these approaches have made relevant contributions, critics have pointed to important limitations that have profound implications for understanding how nature might enhance health and wellbeing and for developing theory, research, policy and design (Menatti and Casado da Rocha, 2016). For the most part, traditional approaches emphasize an anthropocentric perspective which reinforces a notion of nature as separate from humanity, and in extreme cases, merely a resource, commodity or treatment (very much like a “pill”) to be exploited for human benefit (van Heezik and Brymer, 2018). Critics have pointed out that, as a result, these approaches are not able to conceptualize the full range of health enhancing experiences from nature (Brymer et al., 2014; Araújo et al., 2019a) and, importantly, do not fully capture how the person-natural environment system supports health and wellbeing (Hartig et al., 2010; Bratman et al., 2012; Hartig and Jahncke, 2017; Brymer et al., 2020). Furthermore, the predominant anthropocentric focus underlying traditional approaches causes them to largely overlook the inherently multi-dimensional, embedded and embodied complexity of the relationship between humans and nature (Brymer et al., 2014; Conniff and Craig, 2016; Franco et al., 2017; Schweitzer et al., 2018; Araújo et al., 2019b). As a consequence, there is an over-emphasis on structural aspects of the form of nature as an entity, focusing on what nature looks like in terms of color and shape (Brymer et al., 2014). There is also an overemphasis on the individual, focusing on internal processes as the explanation for the person-environment link (Araújo et al., 2019a). More recently, studies examining experiences in nature (Park et al., 2007; Tsunetsugu et al., 2009; Lee et al., 2011; Roe and Aspinall, 2011; Schweitzer et al., 2018) have shown that outcomes may stem from a more ecological, embodied, transactional relationship with the natural environment. The relationship between humans and nature is more complex than traditional theories suggest (Brymer et al., 2019; see also Heft, 2013). In the following section, we present a transactional framework building on Gibson’s (1979) ecological perspective; Ecological Dynamics. This framework which stems from a transactional worldview in which individuals are seen as goal-directed agents whose actions are ongoing and contingent upon a wide range of changing situational factors (Heft, 2012), emphasizes the mutuality of individual-environment relationships as an appropriate theoretical underpinning for understanding the health and wellbeing benefits from the human-natural environment system. Three particular concepts, emanating from the ecological perspective, are highlighted to exemplify the arguments: form of life, affordances and niche construction, suggesting how human-nature interactions might benefit health and wellbeing.

## An Overview of Ecological Dynamics

Ecological Dynamics is a framework that integrates key ideas stemming from ecological psychology and dynamical systems theory and applies them to deepen the understanding of health and wellbeing (Brymer and Davids, 2013, 2014, see Araújo et al., 2020, for a review). Ecological Dynamics has a foundation in the complexity sciences, conceptualizing the individual animal as a complex dynamic system (Kelso, 1995), composed of many interdependent, interacting subsystems or domains (e.g., physical, cognitive, social, emotional). The individual organism forms a part of the larger ecological system. This framework has been employed to interpret behavior in a variety of fields such as health, education, psychology, sport, outdoor education, adventure sports and environmental education (Brymer and Davids, 2013, 2014, 2016; Brymer et al., 2014; Sharma-Brymer et al., 2015; Clough et al., 2016; Davids et al., 2016; Yeh et al., 2016).

Ecological Dynamics takes the person-environment system to be the primary scale of analysis and refutes the dualist assumptions – central to the previously described traditional, interactional (or mechanistic) approaches (see Heft, 2012) – that underpin scientific notions, such as body and mind, or environment and animal. Instead, Ecological Dynamics promotes the holistic acceptance of embeddedness of a person and environment and mind and body (Brymer and Davids, 2013, 2014). The Ecological Dynamics approach further demands reassessment of an inherent “organismic asymmetry”, the intrinsic bias for seeking explanations of human behavior and experiences based on internal mechanisms and referents, regularly promoted in psychological sciences (Dunwoody, 2006; Davids and Araújo, 2010). Behavior is explained as originating from a bounded, self-contained entity and with reference, primarily, to qualities or dispositional properties within an individual. For example, traditional personality psychology perspectives on human-nature relationships typically emphasize the role of specific individual characteristics (e.g., feelings of connection to nature), with little reference to the role of the environment in guiding behaviors. This biased tendency is avoided by considering the mutuality of the person-environment system. Rather than promoting an understanding of behavior as stemming from the mind, in the Ecological Dynamics approach, behavior emerges from the human-environment relationship, the behavior of an animal emerges from its embodiment and embeddedness in an environment.

Another aspect from the traditional environmental focus that can be seen to result from an interactionist (or mechanistic) worldview and is motivated by notions that certain characteristics of place, location and geography (such as Topophilia, place attachment and the notion of therapeutic landscapes) impact health and wellbeing (Dummer, 2008; Menatti and Casado da Rocha, 2016). From this mechanistic worldview, the individual is seen as a bounded and independent entity that exists among independent entities and their influences (Heft, 2012). It is through the interactions between the bounded individual and the bounded natural environment that health and wellbeing is impacted. Typically, this promotes a one-size-fits-all approach where certain environmental characteristics impact, like bouncing billiard balls, the health and wellbeing of the individual. This approach has led to assessments of a “dose response” effect, where exposure to natural environment is required to facilitate good health. The natural environment is treated as a metaphorical “pill” suitable for the treatment of all people from all backgrounds (van Heezik and Brymer, 2018). The environment is perceived as separate from the individual and acting on the individual (Menatti and Casado da Rocha, 2016).

In the context of providing a theoretical underpinning for the human-nature relationship and wellbeing, the Ecological Dynamics perspective takes the individual-environment relationship as the primary scale of analysis. The Ecological Dynamics approach further accepts the observation that individuals have bodies, exist in environments and are constrained by the interacting characteristics of both. Adopting the person-environment system as a scale of analysis for understanding the wellbeing outcomes of human-nature relationships would provide an opportunity to address individual activity and environmental differences that might help explain outcomes and provide a framework for research design.

To understand how this process may occur there are three key conceptual ideas worth highlighting within the Ecological Dynamics framework: *affordances*, *form of life* and *niche construction*. The notion of affordances originated in ecological psychology (Gibson, 1979) and refers to how the environment is perceived in behavioral terms (not in neutral terms like time and space), that is, what the environment offers for doing, and thus combining the nature of the environment with the nature of an individual (Gibson, 1979). In the context of agency, and for the purpose of understanding how the natural environment can motivate and shape health behaviors, affordances can be conceptualized as behavioral invitations offered by the environment, related to the particular capacities, skills and capabilities of the individual (Withagen et al., 2012). That is, in individual environment systems, the behavior which results from the individual-environment link is complex and dynamic; it is not an interpretation of the individual or a response to a stimulus, but an emergent property of this person-natural enviorment system. At any instance in time an innumerable amount of affordances is presented, and available for utilization by an individual. An environment described in terms of affordances changes the emphasis from a structural form description, neutral to the individual, to an active and functional description, in behavioral terms. For example, landscapes traditionally described in terms of color, height, esthetics and so forth can be deemed to consist of behavioral opportunities, such as climbable features, apertures, shelters, flat surfaces, textured or smooth surfaces, inclines, solid or liquid volumes, graspable surfaces, attached objects and so on (Brymer et al., 2014).

Affordances might also be related to cultural and social constraints (Brymer et al., 2014). An individual’s capacity to actualize certain affordances can change over time and through the manipulation of environmental constraints. Importantly, for health and wellbeing, affordances do not always promote positive outcomes and can also invite unhealthy behaviors. For example, our modern-day environment is abounding with convenience – and related – affordances for sedentary and health-threatening behaviors. Think of how city infrastructure promotes car-use over walking or cycling, or how high street lay-out and advertising invite the consumption of fast food (Brymer and Davids, 2016). Conceptually, human beings are adapted to exploit the myriads of affordances available in the human-natural world relationships. Inclusion and removal of certain of these affordances need to be carefully considered in relation to the serious risk of negative unintended consequences for health and wellbeing.

The form of life concept originates from Wittgenstein (1953) and describes how a specific group of human beings or other animals interacts in and with the world around them. That is, form of life describes both the potential and common affordances available in individual-environment systems. This might, for example, manifest as a social or cultural tendency or patterns of behavior (Rietveld and Kiverstein, 2014). For instance, for birds as a form of life, high trees afford shelter and launching pads for flying. However, for monkeys while the same trees might afford shelter, affordances for flying are not available to the monkey form of life. Conceptually, just as monkeys function best in environments appropriate for their adaptations (Gluck and Sackett, 1976), the human form of life is more likely to flourish in the presence of human health and wellbeing affordances.

The terms niche and niche-construction capture an often overlooked, but never-the-less, important aspect of the relevance (and primacy) of the animal-environment scale of analysis and how animal and environments evolve together. That is, both individual and environment are responsible for co-construction and design of affordances. The agency and influence of the individual (or group of individuals) is involved in constructing the everyday environment. Take the beaver that builds the dam in a place that is invited by the relationship between the animal and its environment. The availability of water features and trees appropriate for the particular beaver’s capacities enhance affordances of a certain kind. In a similar vein, humans construct their environment for availability of specific affordances, such as a green park in the city. This co-designing notion extends the evolutionary idea that environments impact the animal, equipping the animal with action capabilities relevant for inhabiting a particular environment. In this way, niche construction supports animal agency and its impact on affordance perception, utilization, creation and destruction (Withagen and van Wermeskerken, 2010). Niche construction not only requires the utilization of affordances, it also consists of a change in the affordance layout. Hence, individuals often create and destroy affordances, with other individuals being exposed to these modified environments as new members of a group. Human beings are both molded by and mold their environment in non-random ways. Over time this mutual co-development can drastically modify behaviors and functions of the animal and the ecosystem, both of which can be inherited by future generations. Thus, the ecological inheritance from one generation to the next encompasses an inheritance of affordances. Geo-physical (including built environments) and social processes can alter the affordances in an individual’s eco-niche. In this way, niche construction can alter the developmental trajectory of a collective system in small or extensive ways, which could be significant (in positive and negative ways) for group health and wellbeing. Conceptually, a form of life that focuses on the realization of affordances, without considering the effect of these affordances on health and wellbeing, might conceivably construct an environment where broader health and wellbeing affordances are depleted. Ecological dynamics predicts that a landscape of nature affordances, lived by a population as their form of life, contributes to increased health and wellbeing, given the embedded and embodied experiences provided by activities in the natural environment according to each individual’s characteristics (Araújo et al., 2019a).

## Implications From the Ecological Dynamics Approach for Understanding and Enhancing Human Health and Wellbeing

The first implication of the Ecological Dynamics approach is the realization that person-environment systems are interdependent. That is, the health outcomes experienced by an individual stem from the relationship between individual skills and characteristics (history, culture, emotions, physiology) and functional environmental characteristics or affordances. From this first animal-environment interdependence implication flows that health and wellbeing interventions and environments need to be designed to provide a wide range of health and wellbeing behavior enrichment affordances (Davids et al., 2016).

The second key implication is that affordances, available through the interaction between humans and natural environments, are richer and more conducive to health and wellbeing outcomes for the human being than heavily manicured urban environments. Practical connotations from this point suggest, for example, that urban design needs to appreciate and provide for key nature affordances, beyond playing fields and picnic areas, that invite a broader range of health and wellbeing behaviors (van Heezik and Brymer, 2018). From a research perspective priority should be placed on determining key affordances in natural contexts. For instance, recent falls prevention research in community dwelling older adults shows that improved gait adaptability is related to a decreased risk of sustaining a gait related fall (van Andel et al., 2018, 2019). This suggests that the design of gait-related activities in environments that include affordances that provoke gait-adaptability, such as those that are available in the natural environment, are needed to provoke adaptable gait and thereby prevent falls in older adults. Ironically, many of the environments constructed for older adults have been designed to take away such affordances. Think of the clinical, low risk environments in traditional nursing homes.

A third implication is that an organism actively molds the environment to better realize certain affordances appropriate for a particular animal, in this case the human animal. However, a potential ramification is that some affordances can be destroyed. Designing environments that maximize affordances for safety, disease minimization, and so on might also destroy affordances for flourishing (health) and empowering experiences (wellbeing). It is therefore important to consider how the construction of human environments maximize affordance potentials for human behaviors, especially health and wellbeing.

In summary, we presented an ecological framework that emphasizes the person-environment relationship as the appropriate scale for analysis and conceptualized it as explanatory for health benefits of human-nature relationships. Three relevant concepts were discussed: form of life, affordances and niche construction, as helpful for appreciating how acting in natural environments might benefit human health and wellbeing. The implications of this approach, from a health and wellbeing perspective, suggest that policy makers and urban designers need to work toward environmental designs that support a landscape of health and wellbeing affordances, that make use of the richness of natural environments.

## Author Contributions

All authors listed have made a substantial, direct and intellectual contribution to the work, and approved it for publication.

## Conflict of Interest

The authors declare that the research was conducted in the absence of any commercial or financial relationships that could be construed as a potential conflict of interest.
